# Autophagy is involved in mouse kidney development and podocyte differentiation regulated by Notch signalling

**DOI:** 10.1111/jcmm.13061

**Published:** 2017-02-03

**Authors:** Chuyue Zhang, Wen Li, Junkai Wen, Zhuo Yang

**Affiliations:** ^1^ School of MedicineState Key Laboratory of Medicinal Chemical BiologyKey Laboratory of Bioactive Materials Ministry of EducationNankai UniversityTianjinChina

**Keywords:** notch signalling, autophagy, differentiation, podocyte, renal development

## Abstract

Podocyte dysfunction results in glomerular diseases accounted for 90% of end‐stage kidney disease. The evolutionarily conserved Notch signalling makes a crucial contribution in podocyte development and function. However, the underlying mechanism of Notch pathway modulating podocyte differentiation remains less obvious. Autophagy, reported to be related with Notch signalling pathways in different animal models, is regarded as a possible participant during podocyte differentiation. Here, we found the dynamic changes of Notch1 were coincided with autophagy: they both increased during kidney development and podocyte differentiation. Intriguingly, when Notch signalling was down‐regulated by DAPT, autophagy was greatly diminished, and differentiation was also impaired. Further, to better understand the relationship between Notch signalling and autophagy in podocyte differentiation, rapamycin was added to enhance autophagy levels in DAPT‐treated cells, and as a result, nephrin was recovered and DAPT‐induced injury was ameliorated. Therefore, we put forward that autophagy is involved in kidney development and podocyte differentiation regulated by Notch signalling.

## Introduction

The morphological development of kidney is from comma to S‐shaped bodies and finally to nephrons that consist of glomerulus, proximal tubule and distal tubule 1. And the formation of the nephron is the key stage in kidney development. Glomerulus is responsible for ultrafiltration of the blood and ensures that necessary plasma proteins are kept. Glomerular filter is composed of podocytes and endothelial cells 2, 3. Podocytes serve as molecular sieves that build the selective permeability properties of the glomerular filter. Thus, podocytes are crucial to exert the function of glomerular. Furthermore, podocyte dysfunction results in the glomerular disease accounted for 90% of end‐stage kidney disease 4. Thus, a deep understanding of the mechanism of glomerular podocyte growth and differentiation is imperative in the process of kidney development and disease.

It is generally known that the function of Notch signalling pathway is to determine cellular differentiation and organ development. There are four Notch receptors in mammals (Notch 1/2/3/4), and they interact with transmembrane ligands of the Delta (Dll1, Dll3 and Dll4) and Jagged (Jag1, Jag2) families. Each of them displays a specific cell and tissue type during development 5, 6, 7. Notch1 is located in the renal epithelial including podocyte component of the mouse metanephros 8. The deletion of Notch2 genes or treatment with a γ‐secretase inhibitor (DAPT) that block Notch signalling pathway in developing kidney leads to metanephroi without glomerular including podocyte precursors and proximal tubules 2. And the expression of Notch3 has been reported in the distal portion of the S‐shaped body 9. Notch4 was mainly detected in endothelial cells 10. Moreover, in developing kidney Notch1 always holds a very high activity in glomerular, but in the mature kidney Notch1 can be detected at very low activity 11. These changes inspired the query: How is the Notch signalling pathway to control kidney development?

Autophagy pathway is the efficient intracellular protein degradation system, and it is essential for maintaining cellular homeostasis 12. The process of autophagy includes that cells devour their cytoplasmic proteins or organelles into double‐membraned structure called autophagosome that subsequently fuse with lysosomes form autophagy–lysosome, finally degradation of the enclosed materials 13, 14. It is controlled by a series of autophagy‐promoting protein act, such as Beclin‐1 is responsible for initiation of autophagosomes 15, LC3 is responsible for the extension and closure of the autophagosome double membranes, and LC3 is conjugated to phosphatidylethanolamine to form lipidated LC3‐II. LC3‐II level reflects the number of autophagosomes and autolysosomes 16. P62 is a polyubiquitin‐binding protein, which is incorporated into the autophagosome and undergoes degradation in autolysosomes, thus inversely related to the autophagy level 17, 18. Autophagy is involved in the pathogenesis of kidney diseases and the physiology of kidney development. Some recent studies have indicated that autophagy can influence glomerular disease by manipulating podocyte homeostasis. Under pathological conditions, loss of autophagy in podocytes results in a dramatically increased susceptibility to various models of glomerular disease. In ageing mice, the podocyte‐specific deletion of autophagy‐related protein 5 would lead to glomerulopathy 19. Under physiological conditions, podocytes exert high levels of autophagy. In addition, in the process of podocyte differentiation, the level of LC3 is a dynamic process 20. The above reports show that autophagy plays a very important role in both podocyte injury and podocyte differentiation. Autophagy is also reported to be related with Notch signalling pathways in different cell models. In the directional differentiation of stem cells, autophagy can regulate Notch degradation, and the inhibition of Notch signalling pathway can also influence the level of autophagy 21, 22. Autophagy can promote the cardiac differentiation of P19CL6 cells by eliminating the Notch intracellular domain (NICD), which promotes transcription of Notch target genes 23. However, little researches have reported about the connection between autophagy and Notch signalling pathway in kidney development including podocyte differentiation from childhood to adulthood in mice. Therefore, our study was designed to test the changes of Notch signalling pathway and autophagy system in the process of kidney development including podocyte differentiation and to study whether Notch signalling pathway regulated kidney development by autophagy.

## Materials and methods

### Antibodies and reagents

N‐[N‐(3,5‐difluorophenacetyl‐L‐alanyl)]‐S‐phenylglycine t‐butyl ester (DAPT) was purchased from Sigma‐Aldrich (CA, USA) (D5942‐5MG). Foetal bovine serum (FBS) and 3‐(4,5‐dimethylthiazol‐2‐yl)‐2, 5‐diphenyltetrazolium bromide (MTT) were bought from Sigma Chemical Co., (St Louis, MO, USA). As for antibodies, anti‐LC3 antibody (M186‐3) was obtained from MBL (Nagoya, Japan) and anti‐β‐actin antibody from Santa Cruz Biotechnology, Inc. (Santa Cruz, CA, USA). Anti‐Beclin‐1 antibody (D40C5) was purchased from Cell Signaling Technology (Danvers, MA, USA). Anti‐Hes‐1 (ab71559), anti‐Notch1 (ab52627), anti‐NICD (ab52301) and anti‐nephrin (ab58968) antibody were bought from Abcam (Cambridge, UK). What needs to be noted is that anti‐Notch1 (ab52627) recognizes both the Notch 1 receptor at the membrane and the proteolytic processing NICD, while the anti‐NICD (ab52301) only recognizes NICD according to Abcam Technology's instructions. The Alexa 594‐conjugated goat antimouse IgG secondary antibody (CA11008S) and 594‐conjugated goat anti‐rabbit IgG secondary antibody (CA11012S) were obtained from Invitrogen (San Diego, CA, USA). The 647‐conjugated goat anti‐rabbit IgG secondary antibody (A21235) was purchased from Life Technologies (Eugene, OR, USA). The DAPI (C0060) was brought from Solarbio Life Science (Beijing, China). The chemiluminescent HRP substrate was purchased from Millipore Corporation (Billerica, MA, USA). All the above antibodies were made in the USA.

### Animals

All animal procedures were accorded to protocols approved by the Animal Care Committee of the Animal Center at the Chinese Academy of Sciences in Shanghai. C57/BL6 mice (20 g, 8–10 weeks of age, both female and male) were provided by the Experimental Animal Center of the Chinese Academy Medical Sciences. Mice were maintained under standard laboratory conditions under artificial 12‐hrs light/12‐hrs dark cycle, and two female mice were paired with one male (2:1) for a period of 5–6 days until mating 24. The birth date of the new born was recorded as P0, and the kidneys of P1, P4, P7, P14, P28 and P49 (mature mice) were collected. The kidneys of same birth date were numbered, and the samples were randomly chosen to perform our experiments. The animal experiments were approved by the Ethics Committee of Nankai University. Studies were designed to minimize the number of animals used and their suffering.

### Cell culture

The conditionally immortalized mouse podocyte clone 5 (MPC5) cells, established by Mundel 25, were widely used 26, 27, 28, 29, 30 and were a kind gift from professor Xiangmei Chen (General Hospital of the People's Liberation Army). The cells harbour a temperature‐sensitive variant of the SV40 large T antigen (tsA58) that is inducible by IFN‐γ and stable at 33°C but rapidly degraded at 37°C 25. At 33°C, the tsA58 allowed for cellular proliferation. The growing MPC5 cells were cultured in RPMI‐1640 media supplemented with 10% FBS, 10 U/ml penicillin and 100 U/ml streptomycin, and 10 U/ml recombinant mouse IFN‐γ. To induce differentiation, MPC5 cells were shifted to 37°C for 2 weeks without IFN‐γ, causing podocytes to become a differentiated phenotype more closely resembling that of podocytes *in vivo* 31.

When shifted to 37°C, podocytes were randomly divided into two groups during the differentiation: the control group and the DAPT‐treated group. These two groups were continued to be divided into three subgroups: undifferentiated podocytes (recorded as differentiating Day 1), differentiating cells (cultured in 37°C for 7 days and recorded as differentiating Day 7) and differentiated ones (cultured in 37°C for 14 days and recorded as differentiating Day 14). DAPT was used to down‐regulate all Notch signalling with the onset of podocyte differentiation (0.5 μM), and lasted for 1, 7 and 14 days, respectively. After differentiation, podocytes were confirmed by the identification of nephrin, a podocyte differentiation marker 31.

### Western blot assay

Western blot assay of renal cortex and podocytes was performed as previously described, and procedures were as follows. They were lysed in lysis buffer [50 mM Tris–HCl (pH 7.4), 150 mM NaCl, 1% Nonidet P‐40, 0.1% sodium dodecyl sulphate (SDS) and 0.5% deoxycholic acid sodium salt (DOC)]. Proteins were extracted for the analysis, and an equal amount of protein loading was separated by SDS‐PAGE on 10% gels, after that transferred to a nitrocellulose membrane. Non‐specific binding sites were blocked by 5% no‐fat milk powder in phosphate‐buffered saline (PBS) with 0.05% Tween‐20 for 2 hrs at room temperature. The membranes were incubated in anti‐LC3, anti‐Hes‐1, anti‐β‐actin, anti‐nephrin, anti‐Notch1, anti‐Beclin‐1 and anti‐NICD antibodies, respectively. After 12 hrs of incubation at 4°C, the membranes were washed in TBST for 10 min. ×4, followed by incubation with anti‐rabbit IgG (H+L), HRP conjugate [W4011; Promega (Madison, USA), working dilution 1:3000] or antimouse IgG (H+L) HRP conjugate (W4021; Promega, working dilution 1:3000) for 1 hrs at room temperature. All experiments were performed at least three times. For analysis, quantitative analyses were achieved by scanning and determining the intensity of the hybridization signals. IMAGEJ software was used to evaluate the differences between the sample of interest and the β‐actin antibodies, respectively.

### Immunofluorescence staining

#### Immunofluorescence staining of kidney samples

Immunofluorescence analyses were performed as previously described 3. Briefly, the kidneys were obtained from C57BL6 mice at indicated ages, fixed in 4% paraformaldehyde in PBS for 4 hrs at 4°C, subjected to 30% sucrose overnight at 4°C, embedded in OCT compound (Tissue‐Tek, Miles) and sectioned at 6 μm (Leica CM1850; Leica Instruments, Wetzlar, Germany). Slides were washed 3 × 5 min. in PBS and blocked with 10% serum of the secondary antibody host species for 1 hr. And then slides were incubated with primary antibodies at 4°C overnight as follows: Notch1, Beclin‐1, LC3 and nephrin. Dilution and other supplementary information of the primary and secondary antibodies are as follows.

Dilution of primary antibodies:
Notch1 (ab52627; Abcam): 1:500;
LC3 (M186‐3; MBL): 1:500
P62 (ab56416): 1:500

The secondary antibodies:
The Alexa 488‐conjugated goat antimouse IgG secondary antibody (CA11008S; Invitrogen) and 647‐conjugated goat anti‐rabbit IgG secondary antibody (A21235; Life Technologies) as well as the Alexa 594‐conjugated goat antimouse IgG secondary antibody (CA11008S) were used at 1:1000.

Imaging was conducted with a confocal microscope (Olympus FV1000; Tokyo, Japan), and the fluorescent intensity was quantified using IMAGEJ software (National Institutes of Health, Bethesda, MD, USA).

#### Immunofluorescence staining of podocytes

The three various differentiating stages of MPC5 cells were treated with DAPT (0.5 μM) along with the differentiation for 1 day, 7 days and 14 days, respectively, which were named as differentiating Day 1, Day 7 and Day 14. After fixation in 4% paraformaldehyde for half an hour, cells were washed with PBS 5 min. ×3 and permeabilized with 0.5% Triton X‐100 and then they were blocked with 10% NGS for 2 hrs at room temperature. Following that, podocytes were incubated with anti‐LC3, anti‐NICD and anti‐nephrin antibodies and thereafter, washed with PBS, and MPC5 cells were incubated with the Alexa 594‐conjugated goat antimouse IgG secondary antibody (1:1000) and 647‐conjugated goat anti‐rabbit IgG secondary antibody (1:1000). Subsequently, the podocyte nuclei were stained by DAPI. Samples were examined under a fluorescence microscope (Olympus FV1000, Japan), and the fluorescent intensity was quantified as previously reported 32 using IMAGEJ software.

### Statistical analysis

Sample size was in accordance with previous studies for animal experiments 24, 33. All data were expressed as mean ± S.D. and analysed by GRAPHPAD PRISM 5.0 software (GraphPad Software, Inc., La Jolla, CA, USA) and SPSS 17.0 software (SPSS, Inc., Chicago, IL, USA). There was a minimum of six animals per age group. Results were analysed by a one‐way anova followed by the LSD *post hoc* test, and statistical significance was defined as *P* < 0.05. All experiments were performed at least in triplicate.

## Results

### Nephrin expression increased from post‐natal Day 1 to adulthood during renal development

**Figure 1:**
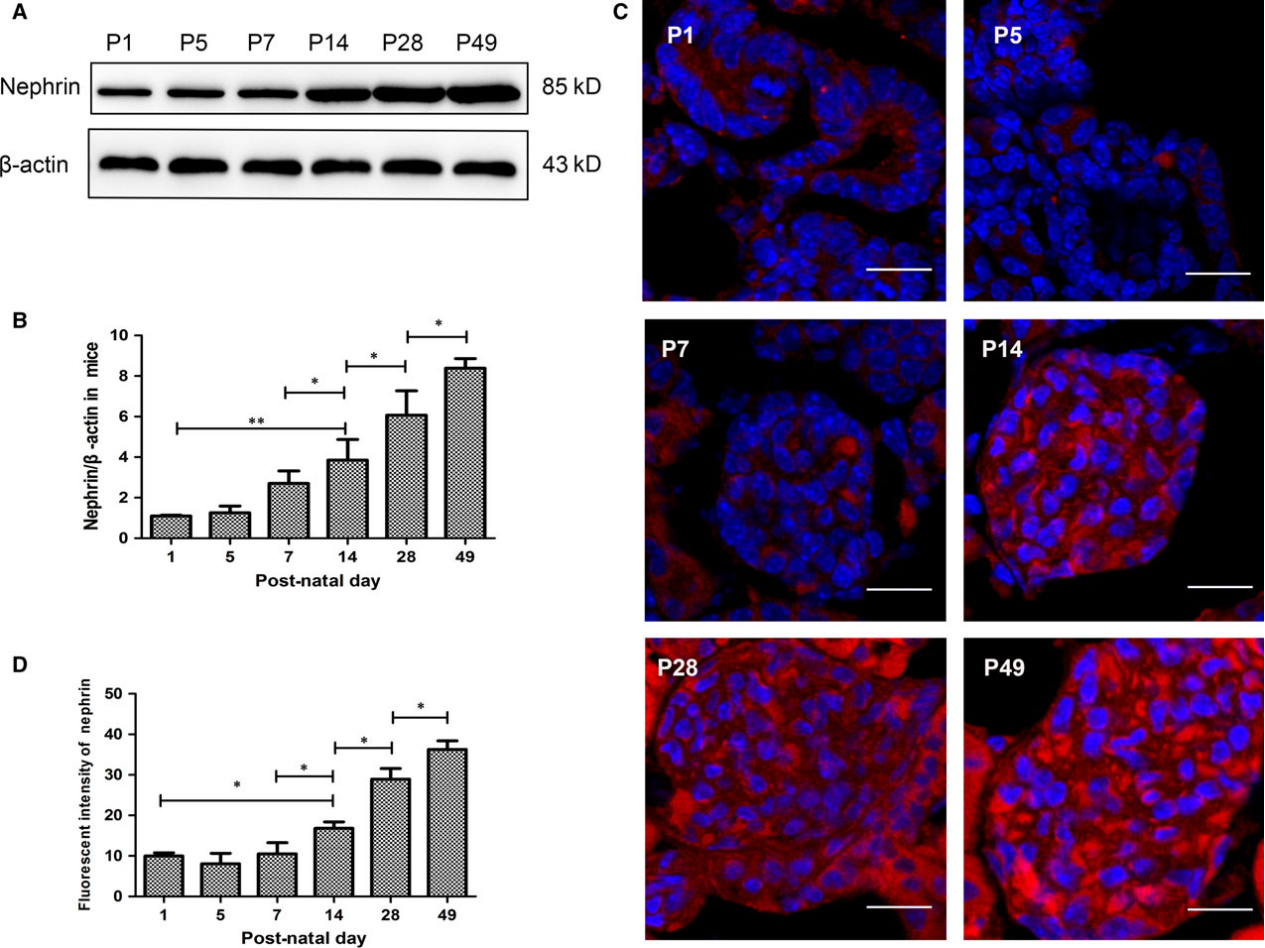
Nephrin increased from post‐natal Day 1 to adulthood during renal development. (**A**) Nephrin expression was assessed by Western blot assay on post‐natal day 1(P1), P5, P7, P14, P28 and P49 in renal cortex during kidney development. (**B**) Quantitative determination of Western blot assays for nephrin as beta‐actin was a consult. (**C**) Immunofluorescence staining of nephrin (Alexa 594, red) in different development stages. Scale bar: 30 μm. (**D**) Fluorescent intensity of nephrin in glomeruli. Data were presented as the means ± S.D. *n* = 6/group. *P < 0.05.

In our experiment, nephrin expression was detected in different developmental stages to identify the degree of renal development as well as podocyte maturation. The birth date of mice was recorded as post‐natal Day 0 (P0), and kidneys of P1, P5, P7, P14, P28 and P49 (mature mice) were collected and renal cortex was separated, where the podocytes were localized. It was observed that nephrin increased all the way through the renal development, from P1 to adulthood (Fig. 1A and B), marking the accumulation of mature podocytes. To label the podocytes more intuitionistic, immunocytofluorescence assay was used and the results are shown in Figure 1C, which was consistent with the conclusion in Western blot assay.

### The dynamic expression of Notch1 coincided with the autophagy activity during renal development: peaked between P7 and P14 and reduced in adulthood

**Figure 2:**
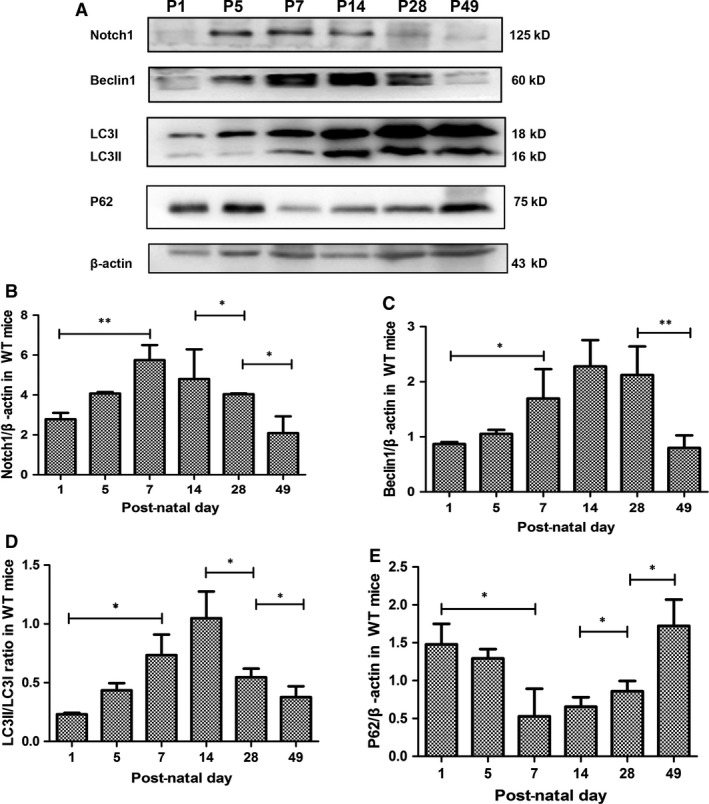
The dynamic changes of Notch1 coincided with the autophagy during renal development. (**A**) Immunoblot analysis of Notch1 and autophagy in renal cortex in post‐natal day 1 (P1), P5, P7, P14, P28 and P49. (**B**) Quantitative immunoblot analysis of the full‐length Notch1. (**C–E**) Summarized data from densitometric analysis of the Beclin‐1, LC3 and P62 signals from immunoblots, respectively. Data were presented as means ± S.D. of ≥3 independent experiments. *P < 0.05, **P < 0.01

To find out how Notch1 functions during the renal development as well as the podocyte differentiation, we first examined the Notch1 levels (full‐length Notch1) in kidneys of P1, P5, P7, P14, P28 and P49. The Notch1 bands are shown in Figure 2A, as the quantitative immunoblot analysis showing below (Fig. 2B). The autophagy levels were assessed by detecting Beclin‐1, the ratio of LC3‐II/LC3‐I 34 and P62 (Fig. 2A, C–E). The analyses indicated that the changing level of autophagy was similar to Notch1: first, an uptrend from P1 to P7, then a sharp decline between P14 and P49 and the peak between P7 and P14.

**Figure 3:**
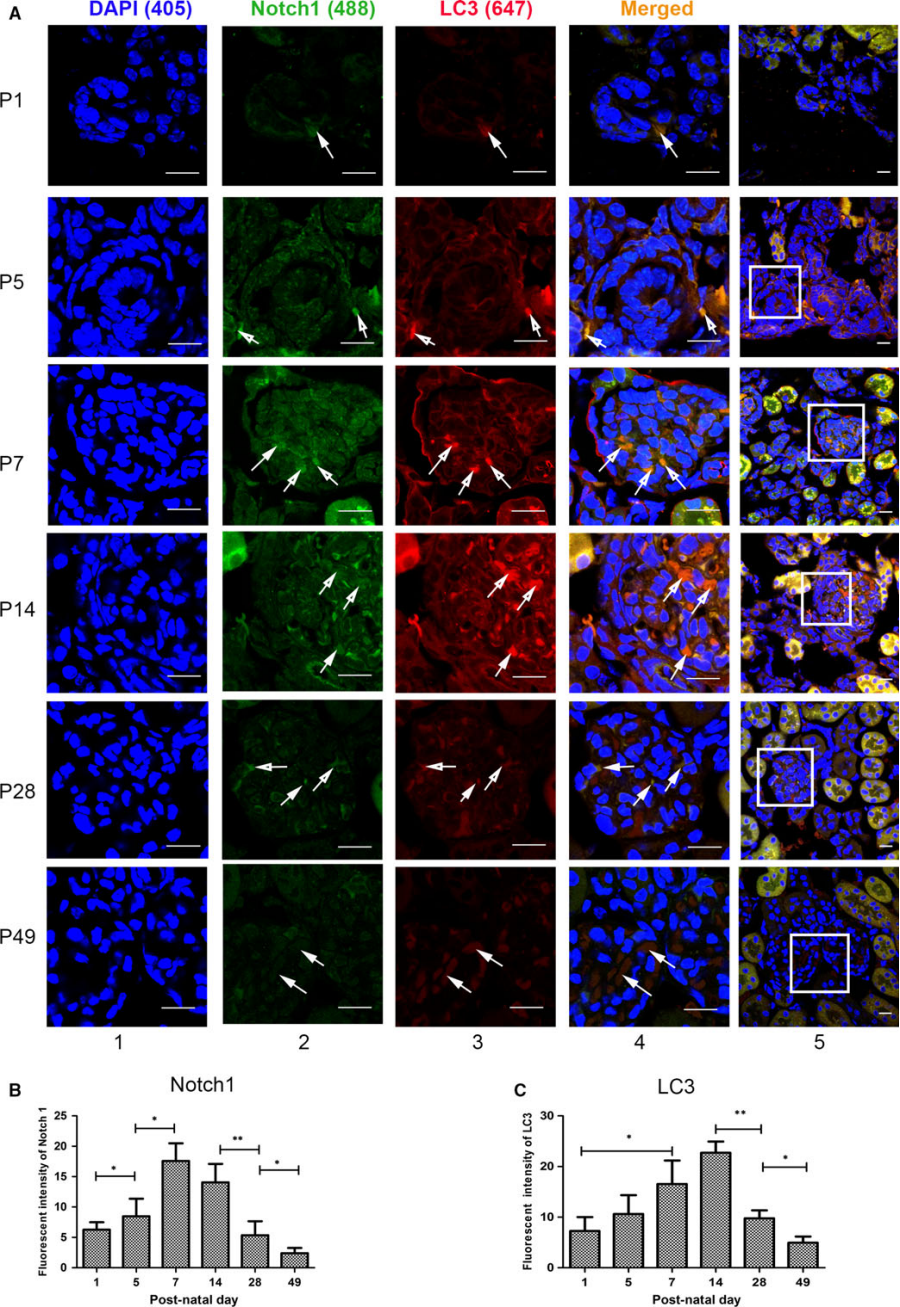
Notch1 levels were in line with the trend of autophagy reflected by LC3 during renal development in immunofluorescence staining. (**A**) The morphological evaluation of Notch1 (Alexa 488, green) and LC3 (Alexa 647, red) on P1, P5, P7, P14, P28 and P49 in glomeruli during renal development. The arrows show LC3 and Notch1 colocalization. Scale bar: lane 1, 10 μm; lane 5, 30 μm. (**B–C**) Fluorescent intensity of Notch1 and LC3 in glomeruli, respectively. Data were presented as the means ± S.D. of ≥3 independent experiments. *P < 0.05.

**Figure 4:**
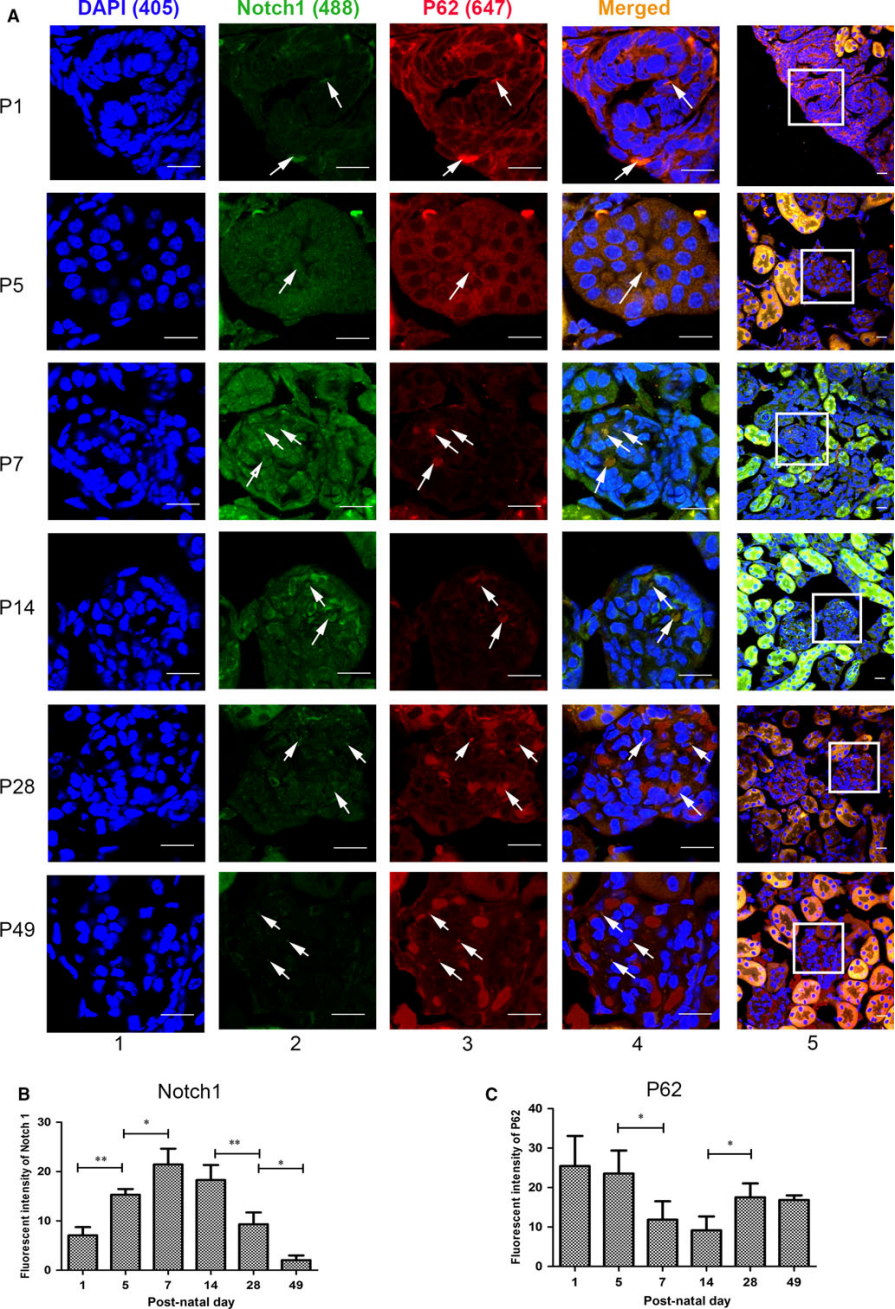
Notch1 levels coincided with the autophagy status inversely reflected by P62 during renal development in immunofluorescence staining. (**A**) The morphological evaluation of Notch1 (Alexa 488, green) and P62 (Alexa 647, red) on P1, P5, P7, P14, P28 and P49 in glomeruli during renal development. The arrows show P62 and Notch1 colocalization. Scale bar: lane 1, 10 μm; lane 5, 30 μm. (**B–C**) Fluorescent intensity of Notch1 and P62 in glomeruli, respectively. Data were presented as the means ± S.D. of ≥3 independent experiments. *P < 0.05.

Double immunofluorescence staining analysis was conducted with LC3 and Notch1 (Fig. 3), together with P62 and Notch1 (Fig. 4), which provides a second way to explore the correlation between Notch1 and autophagy, which also disclosed that dynamic expression of Notch1 resembled the autophagy levels in renal cortex during kidney development. To study the direct relations of them, and how they affect podocytes, further *in vitro* experiments were as follows.

### Notch1 signalling expressed during the differentiation process, and became almost silent when podocytes were differentiated

To identify how Notch pathway directly affects podocyte maturation, the MPC5 cells were cultured and three differentiated stages were studied: podocytes of undifferentiated, differentiating and differentiated were recorded as differentiating days 1, 7 and 14, respectively. As γ‐secretase cleavage of Notch generates NICD, which was the NICD, NICD represented for Notch activation. Therefore, NICD and Hes‐1, the typical downstream protein of Notch1 signalling, were detected within podocyte differentiation.

**Figure 5:**
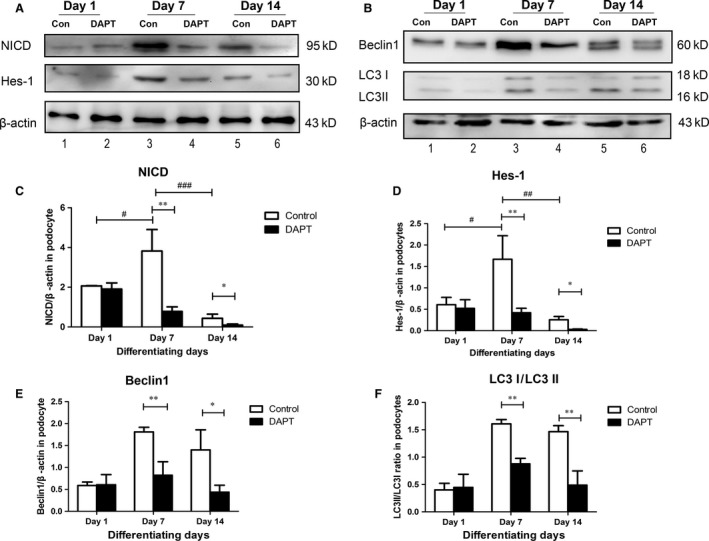
Down‐regulation of all Notch signalling decreased autophagy levels within podocyte differentiation. (**A**) Analyses of Notch pathway by immunoblotting. (**B**) Analyses of autophagy by immunoblotting. (**C–F**) Quantitative determination for NICD, Hes1, Beclin‐1 and LC3, relative to actin, normalized for relevant control. Data were presented as means ± S.D. of ≥3 independent experiments. *P < 0.05, **P < 0.01, #*P* < 0.05, ##*P* < 0.01, ###*P* < 0.001.

It was showed that both NICD and Hes‐1 accumulated mostly in differentiating podocytes (Fig. 5A, lane 3 and Fig. 5C and D), much more than that of undifferentiated cells (Fig. 5A, lane 1 and Fig. 5C and D) and mature podocytes (Fig. 5A, lane 5 and Fig. 5C and D). This observation indicated that Notch1 signalling expressed mostly during the differentiation process, and became almost silent when differentiated, which was in line with the report that Notch pathway became silent in mature glomeruli 35.

### The autophagy levels decreased after persistent down‐regulation of Notch signalling during podocyte differentiation

To figure out the underlying mechanism by which Notch pathway influenced podocytes, Notch pathway was down‐regulated using DAPT during the entire differentiation process (0.5 μM). DAPT was a γ‐secretase inhibitor, which could block all Notch signalling 36. Both Notch expression and autophagy were tested in control and DAPT‐treated cells within the specification.

In the control group, little Beclin‐1 expression can be recognized in undifferentiated MPC5 cells (Fig. 5B, lane 1 and Fig. 5E), and it significantly accumulated on differentiating cells (Fig. 5B, lane 3), while declined when podocytes became mature after differentiating for 14 days. In agreement with this, the ratio of LC3‐II/LC3‐I exhibited the same trend (Fig. 5B and F), indicating that autophagy activity played an essential role within the maturation of podocytes, which was coincided with Notch1 signalling.

Then, we down‐regulated all the Notch signalling by DAPT, and the efficiency of DAPT was demonstrated by decreased expression of NICD and Hes‐1 (Fig. 5A, lanes 2, 4 and 6; Fig. 5C and D). Under DAPT treatment along with cellular differentiation, autophagy levels fell down evidently on the 7th differentiating day, as seen in bands of Beclin‐1 and the ratio of LC3‐II/LC3‐I (Fig. 5B, lane 4 and Fig. 5E and F), and autophagy continued to decline when cells were mature (Fig. 5B, lane 6 and Fig. 5E and F).

**Figure 6:**
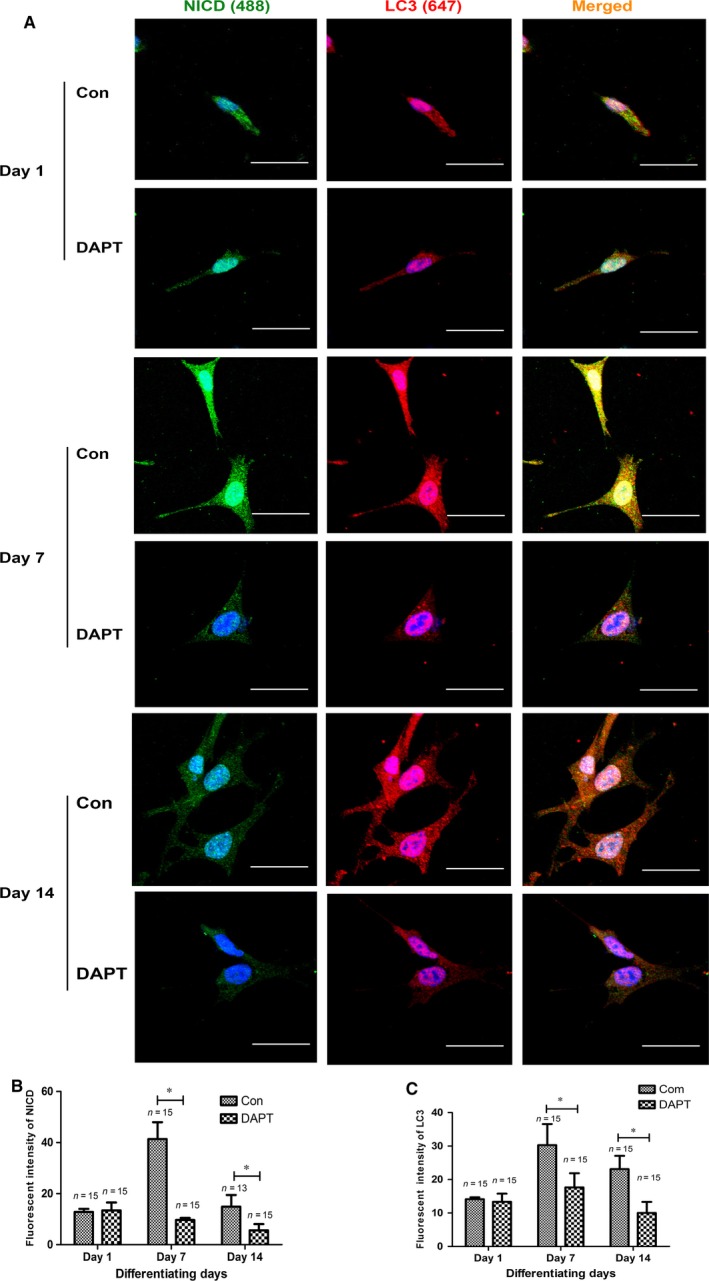
The colocalization of NICD and autophagy in control and DAPT‐treated podocytes during the differentiation process. (**A**) NICD (Alexa 488, green) and LC3 (Alexa 647, red) were evaluated in undifferentiated (Day 1), differentiating (Day 7) and differentiated cells (Day 14). Scale bar: 40 μm. (**B–C**) Fluorescent intensity of NICD and LC3 in podocytes at different differentiation stages. Data were presented as the means ± S.D. of ≥3 independent experiments. *P < 0.05.

In support of this notion, double staining with anti‐NICD and anti‐LC3 antibodies was performed (Fig. 6). It was found that podocytes in the presence of DAPT contained less autophagosomes than that of normal podocytes during the differentiation process. Taken together, autophagy levels decreased after prolonged down‐regulation of Notch signalling (*via* DAPT) during the maturation of podocytes.

### Persistent down‐regulation of Notch signalling diminished, but not completely eliminated differentiation marker nephrin in podocytes

**Figure 7:**
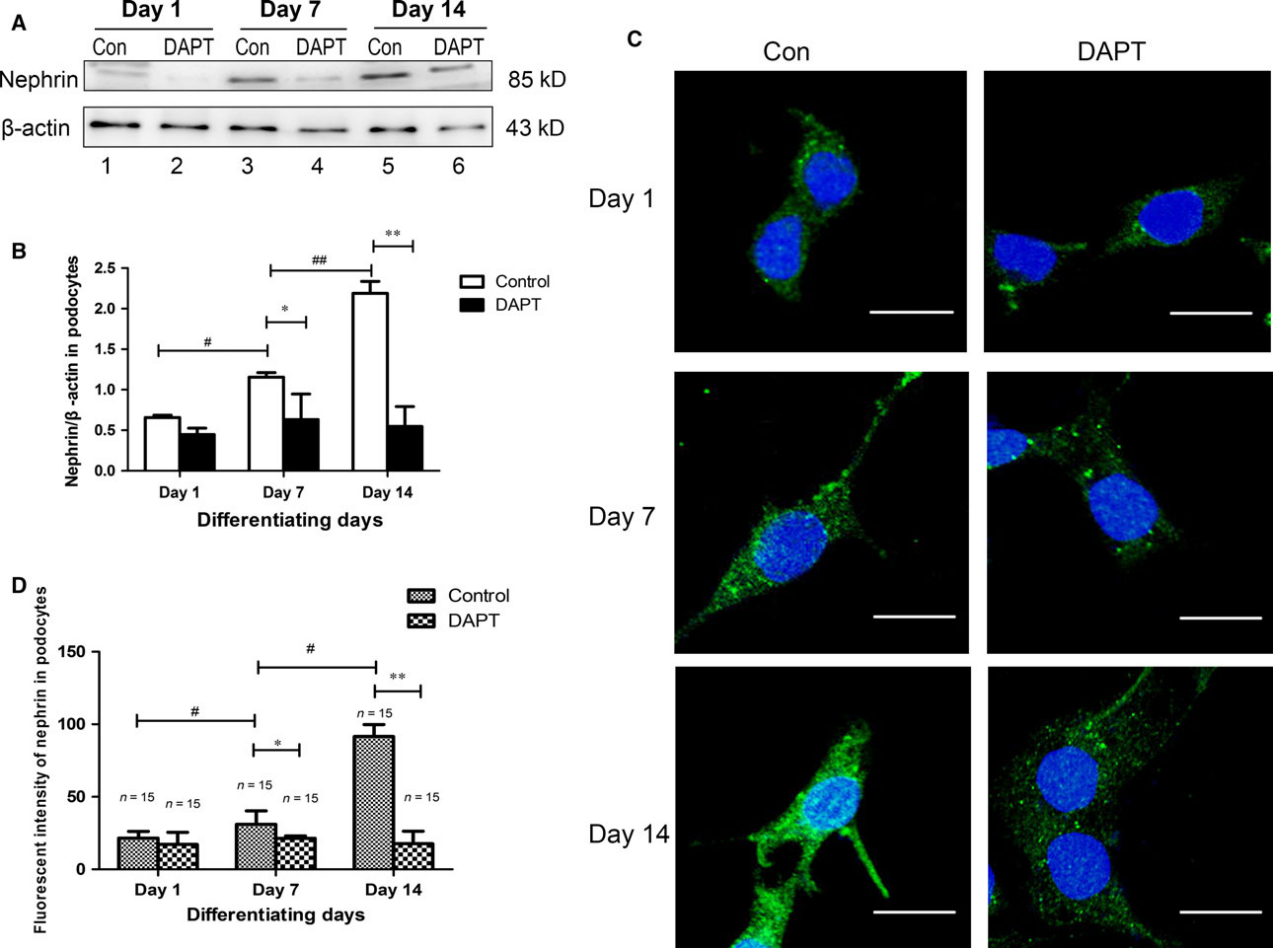
Persistent down‐regulation of Notch signalling diminished podocyte‐specific protein nephrin along with the differentiation. (**A**) Nephrin expression was assessed by Western blot assay at control and DAPT‐treated MPC5 cells on different development stages. (**B**) Quantitative immunoblot analysis of nephrin with beta‐actin as a control. (**C**) Representative confocal microscopic images of nephrin (Alexa 488, green). Scale bar: 40 μm. (**D**) Fluorescent intensity of nephrin in podocytes at different development stages. Data were presented as the means ± S.D. of ≥3 independent experiments. *P < 0.05.

To further study the outcome of DAPT‐induced autophagic reduction, the podocyte‐specific protein nephrin was detected. As seen in control cells, nephrin expression was enhanced as podocytes differentiated, and reached a plateau on Day 14, marking the maturation (Fig. 7A, lanes 1, 3 and 5; Fig. 7B). Nevertheless, the nephrin expression diminished along with the differentiation, after exposure to DAPT during the differentiation period for differentiating cells (Fig. 7A, lanes 3–4; s.7B) and fully differentiated podocytes (Fig. 7A, lanes 5–6; Fig. 7B), respectively.

Immunocytofluorescence assay was also performed and nephrin was stained (Fig. 6C and D). In the control group, when MPC5 cells were differentiated for 7 days (37°C, IFN‐γ), spindle‐like projections were observed, and while differentiated for 14 days, large, flat and arborized cells were seen with more prominences. When DAPT down‐regulated Notch pathway, the staining with anti‐nephrin was still observed during the specification (Day 7 and Day 14); however, the fluorescence intensity decreased and numbers of bright fluorescent particles in cells diminished, and the podocyte‐specific foot processes were not dominated. It is tempting to speculate that down‐regulation of Notch signalling diminished, but does not completely prevent podocyte differentiation.

### Enhancing autophagy levels ameliorated DAPT‐induced injury

In the section above, we speculated that Notch inhibition in podocytes decreased autophagy levels and diminished differentiation marker nephrin. Further, we enhanced the autophagy level by rapamycin, which is a mTORC1 inhibitor and inducer of autophagy 37, 38, 39, 40, 41, 42, to see whether the injury in differentiation would be rescued.

**Figure 8:**
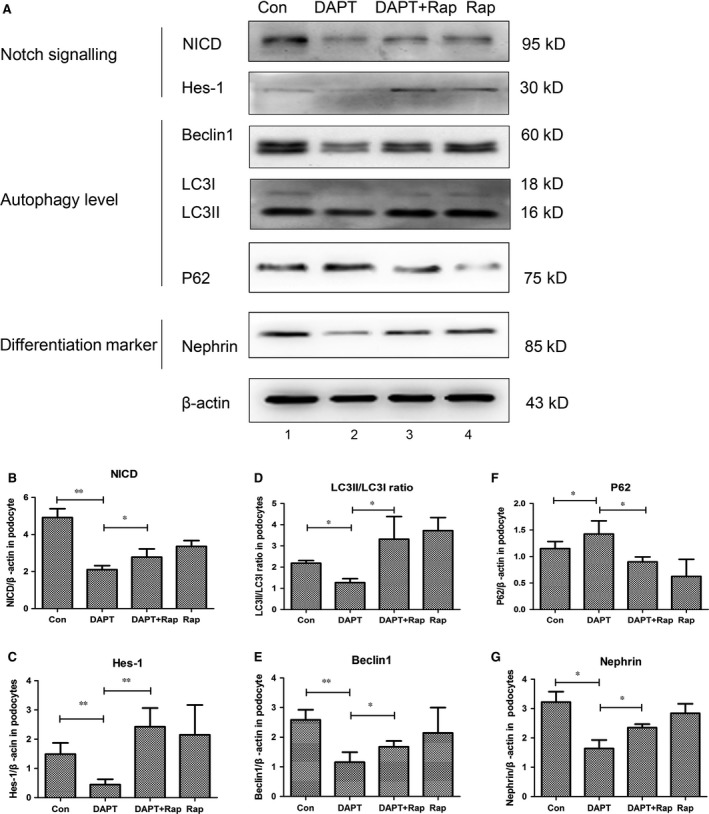
Enhancing autophagy levels ameliorated DAPT‐induced injury. (**A**) The mouse podocyte clone 5 (MPC5) cells were cultured and rapamycin (10 ng/ml) was added to enhance autophagy levels. The Western blot results of Notch signalling (NICD and Hes‐1), autophagy levels (Beclin‐1, LC3II/I ratio and P62) and differentiation marker (nephrin) were showed, relative to actin, normalized for relevant control. (**B‐C**) Quantification of Notch signalling NICD and Hes‐1 in immunoblots, respectively. (**D–F**) Densitometric analysis of the autophagic marker Beclin‐1, LC3 II/I ratio and P62 in immunoblots, respectively. (**G**) Quantitative determination of differentiation marker nephrin in Western blot assays. Data were presented as means ± S.D. of ≥3 independent experiments. *P < 0.05, **P < 0.01.

The MPC5 cells were incubated in the medium without DAPT, containing DAPT (0.5 μM), DAPT (0.5 μM) + rapamycin (10 ng/ml) and rapamycin (10 ng/ml) and differentiated for 7 days (as differentiating cells). In DAPT‐treated cells, autophagy status that reflected by Beclin‐1, LC3II/I ratio and P62 was augmented by rapamycin (Fig. 8A, lanes 2–3; Fig. 8D and F).

Molecules of Notch signalling were decreased by DAPT (Fig. 8A, lanes 1–2; Fig. 8B and C); however, when rapamycin was added in DAPT (consequently autophagy levels were increased), the DAPT‐reduced Notch signalling was enhanced compared with that of DAPT group (Fig. 8A, lanes 2–3; Fig. 8B and C).

As to podocyte differentiation marker nephrin, it was diminished when Notch signalling was down‐regulated by DAPT (Fig. 8A, lanes 1–2; Fig. 8G), but the expression remarkably increased after exposure to both DAPT and rapamycin (Fig. 8A, lanes 2–3; Fig. 8G), and in other words, enhancing autophagy levels ameliorated DAPT‐induced injury in podocytes. Therefore, we put forward that autophagy was one of the mechanisms by which Notch signalling regulated podocyte differentiation.

## Discussion

Notch signalling is an evolutionarily conserved cell–cell communication 43, which regulates the implementation of differentiation, proliferation and apoptosis. Thus, it makes a crucial contribution in kidney development including podocyte specification 9, 11, 44, 45. In early differentiation of podocytes, Cheng demonstrated an important role for Notch signalling 2. The cultivation of mouse metanephroi with γ‐secretase inhibitor DAPT (the blocker of all Notch pathways) caused a severe deficiency in glomeruli, for renal epithelia were unable to give rise to podocytes. In support of this notion, a report showed 46 that mature glomeruli did not form in mice with genetic deletion for γ‐secretase complex. Interestingly, molecular of Notch signalling progressively diminished when kidney as well as podocytes became mature 9, 11, 47, and Notch pathway seemed to be dispensable for podocytes in an advanced stage of differentiation 48, 49. Collectively, Notch pathway is essential in podocyte differentiation, and we began to wonder the mechanism underlying Notch regulation.

When we study the underlying mechanism by which Notch pathway modulates podocyte differentiation during renal development, we found reports illustrating that autophagy was related with Notch signalling pathway 21, 50, 51, 52. However, until recently little was known about the correlation between Notch signalling and autophagy during renal development especially in podocyte differentiation, so we examined the autophagy levels and Notch1 expression in developing kidney. According to the data (Figs 2, 3 and 4), the dynamic expression of Notch1 was coincided with autophagy activity during renal development. Thus, both Notch1 and autophagy contributed a lot within renal development as well as podocyte differentiation, and their similar dynamic changes suggested a correlation between them.

To better demonstrate their correlation, MPC5 cells were cultured, and all Notch pathways were down‐regulated by DAPT, the inhibitor of all Notch signalling 53 along with the cellular differentiation. It showed that down‐regulation of Notch signalling decreased autophagy levels in podocyte maturation. So far, we have proved that Notch pathway can regulate autophagy within podocyte differentiation.

Autophagy is increasingly recognized for the important role in specific cytosolic rearrangements needed for proliferation and differentiation during embryogenesis and post‐natal development 54, 55, 56, 57, 58, 59, 60, 61 including podocyte maturation 20. When autophagy activity was deficient, cellular homeostasis was impaired 62, 63, 64, 65 and cellular integrity could not be maintained 19, even maturation could be delayed 66, so when autophagy levels were decrease by Notch inhibition (Figs 5 and 6), podocyte differentiation was impaired, which was verified by the decline of podocyte differentiation marker nephrin (Fig. 7). To better understand the relationship between Notch signalling and autophagy in podocyte differentiation, we added rapamycin to enhance autophagy levels, and as a result, nephrin was recovered and DAPT‐induced injury was ameliorated. This result disclosed that the differentiation defect was directly caused by autophagy reduction, and increasing autophagy status can rescue the differentiation when Notch signalling was impaired.

In conclusion, autophagy is not only involved in widely reported disease models 12, 67, but also crucial in renal development, and we put forward that autophagy is involved in podocyte maturation that regulated by Notch pathway, and when Notch signalling is impaired, enhancing autophagy can rescue podocyte differentiation. Our findings open up new perspectives for autophagy in kidney development, and the crosstalk between autophagy and Notch pathway in renal development needs further exploring.

## Author contribution

ZY conceived and designed the experiments. CYZ, WL and JKW performed the experiments. CYZ and WL analysed the data. CYZ, WL and JKW contributed to reagents/materials/analysis tools. CYZ, WL and ZY wrote and edited the manuscript.

## Competing interests

The authors have declared that no competing interest exists.
